# The regulation and deregulation of Wnt signaling by PARK genes in health and disease

**DOI:** 10.1093/jmcb/mjt037

**Published:** 2013-10-09

**Authors:** Daniel C. Berwick, Kirsten Harvey

**Affiliations:** Department of Pharmacology, UCL School of Pharmacy, University College London, 29-39 Brunswick Square, London WC1N 1AX, UK

**Keywords:** Wnt signaling, neurodegeneration, Parkinson's disease, genetics of Parkinson's disease, LRRK2, treatment

## Abstract

Wingless/Int (Wnt) signaling pathways are signal transduction mechanisms that have been widely studied in the field of embryogenesis. Recent work has established a critical role for these pathways in brain development, especially of midbrain dopaminergic neurones. However, the fundamental importance of Wnt signaling for the normal function of mature neurones in the adult central nervous system has also lately been demonstrated by an increasing number of studies. Parkinson's disease (PD) is the second most prevalent neurodegenerative disease worldwide and is currently incurable. This debilitating disease is characterized by the progressive loss of a subset of midbrain dopaminergic neurones in the *substantia nigra* leading to typical extrapyramidal motor symptoms. The aetiology of PD is poorly understood but work performed over the last two decades has identified a growing number of genetic defects that underlie this condition. Here we review a growing body of data connecting genes implicated in PD—most notably the *PARK* genes—with Wnt signaling. These observations provide clues to the normal function of these proteins in healthy neurones and suggest that deregulated Wnt signaling might be a frequent pathomechanism leading to PD. These observations have implications for the pathogenesis and treatment of neurodegenerative diseases in general.

## Introduction

Parkinson's disease (PD) is a progressive neurodegenerative disease first described in 1817 as a ‘shaking palsy’ (Parkinson, 2002). Individuals with PD typically present with motor dysfunction—the four cardinal symptoms being resting tremor, bradykinesia, rigidity, and postural instability—although additional non-motor symptoms such as dementia and depression are very common (Dauer and Przedborski, 2003; Gasser, 2009). Pathologically, post-mortem brains from PD patients display two hallmarks. First, the presence of Lewy bodies—proteinaceous deposits rich in α-synuclein protein—and second, the loss of dopaminergic (dopamine producing) neurones of the *substantia nigra pars compacta* (Dauer and Przedborski, 2003; Gasser, 2009). However, despite nearly 200 years of study PD is still incurable, with the molecular events underlying the aetiology and progression of the disease remaining stubbornly hidden. Treatments for PD remain focused on alleviating symptoms and the most common therapeutic strategy, pharmacological replacement of lost dopamine, was first described over 40 years ago (Cotzias et al., 1969). Since the major risk factor for PD is age, the increased life expectancies observed across much of the world mean that PD will place a large burden on healthcare systems in years to come. As such, the development of novel treatments for PD—most importantly drugs that cure or at least arrest disease progression—is of imperative clinical need. Thus an understanding of the molecular mechanisms underlying PD is urgently required.

In this article we summarize recent data from the study of genes linked to PD that suggest a central importance of Wingless/Int (Wnt) signaling pathways for the normal function of dopaminergic neurones. These hereditary clues indicate that perturbation of these cell signaling cascades might represent a key event in the early pathogenesis of PD. Deregulation of Wnt signaling has the potential to explain many of the commonly observed features of neurodegeneration, while the sheer number of genes implicated in PD that are also involved in Wnt signaling is compelling. In light of the vital requirement for improved PD treatments and the therapeutic potential of targeting signal transduction cascades, these connections between PD and Wnt signaling make a persuasive and timely case for research into the role of Wnt pathways in the adult brain, both in health and disease.

## The canonical Wnt pathway

Wnt signaling pathways are a group of highly conserved cell signaling cascades with a well-established importance to animal development and cancer biology (Maiese et al., 2008; Freese et al., 2010). There are generally considered to be three major branches of Wnt signaling: the so-called canonical (or Wnt/β-catenin), planar cell polarity (PCP), and Wnt/calcium (Wnt/Ca^2+^) cascades. This article will focus on the canonical pathway since the majority of research reviewed focuses on this signaling cascade. Nonetheless, it should be stressed that roles for the PCP and Wnt/Ca^2+^ pathways in the pathogenesis of PD cannot be excluded.

The term Wnt describes the secreted ligand proteins that bind to the extracellular domains of frizzled (FZD) receptors on target cells. Wnt ligands are usually glycosylated and are relatively large for secreted morphogens, suggesting that the sites of secretion and action tend to be relatively close (i.e. Wnt ligands are predominantly autocrine and paracrine, rather than endocrine). In humans there are 19 genes encoding Wnt ligands and 10 genes encoding FZD receptors (Wnt home page: http://www.stanford.edu/group/nusselab/cgi-bin/wnt/). Further signaling complexity is conferred by membrane co-receptors. In the case of the canonical Wnt pathway, these receptors are low density lipoprotein receptor-related proteins 5 and 6 (LRP5/6). The binding of Wnt ligands to the combination of FZD and LRP5/6 proteins allows the extracellular signal to be relayed across the plasma membrane, leading to the activation of intracellular signaling.

**Figure 1:**
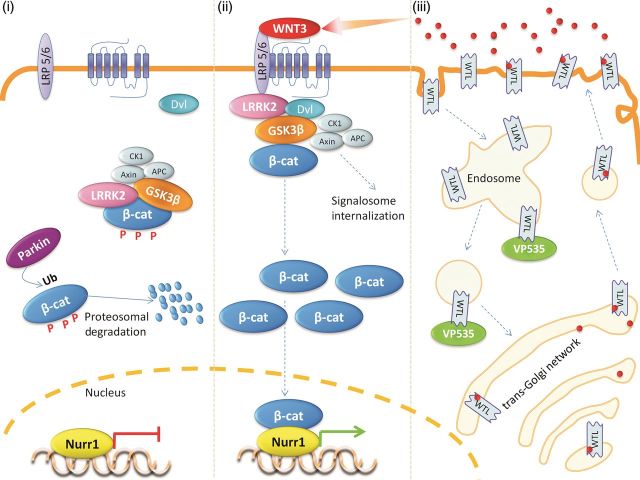
Potential sites of canonical Wnt signaling deregulation in PD. As outlined in the main text, six proteins—GSK3β, LRRK2, Nurr1, Parkin, VPS35, and WNT3—have been linked to canonical Wnt signaling and genetic risk of PD. The sites where these proteins affect the canonical pathway are represented graphically. The diagram is divided into three sections; from left-to-right these are (i) basal canonical Wnt signaling, (ii) activated Wnt signaling, and (iii) Wnt secretion. (i) In the basal state, β-catenin, the main effector of the canonical Wnt pathway, is repressed by GSK3β, LRRK2, and Parkin. GSK3β phosphorylates β-catenin, triggering the ubiquitination (Ub) of this protein by Parkin, leading to the proteosomal degradation of β-catenin. (ii) Upon binding of Wnt ligand such as WNT3, LRRK2, β-catenin, GSK3β, and associated proteins are recruited to membrane receptor complexes. The phosphorylation of β-catenin by GSK3β is repressed, stabilizing β-catenin and allowing this protein to enter the nucleus. Nuclear β-catenin binds and transactivates target transcription factors including Nurr1, driving the expression of downstream genes. In both basal and activated conditions LRRK2 functions as a scaffolding protein. (iii) Wnt ligand secretion is mediated by Wntless (WTL), which transports Wnt ligands from the *trans*-Golgi network to the plasma membrane where Wnts are released. This process is dependent on the recycling of Wntless back from the plasma membrane, via early endosomes to the *trans*-Golgi network. This last step is mediated by the retromer complex, of which VPS35 is an essential component. Loss of VPS35 function leads to an accumulation of Wntless in the endosomal system, and decreased secretion of Wnt ligands.

The classical output of the canonical Wnt pathway is the activation and nuclear recruitment of β-catenin (hence the alternative name, Wnt/β-catenin signaling). The canonical pathway is unusual as several events take place in the absence of a stimulus. In the basal state, β-catenin is sequestered into an inhibitory cytosolic complex known as the β-catenin destruction complex. Within this complex β-catenin is phosphorylated by glycogen synthase kinase 3β (GSK3β), resulting in the targeting of β-catenin for proteosomal degradation. Thus, in the absence of stimulation by Wnt ligand, β-catenin is continually degraded. The activation of FZD receptors causes the recruitment of dishevelled (DVL) proteins to the plasma membrane, where DVLs are thought to associate with the intracellular surface of FZD receptors. Through additional interaction with components of the β-catenin destruction complex, DVL proteins mediate the relocalization of the β-catenin destruction complex to juxtamembrane sites. The resultant formation of large membrane-spanning complexes, containing extracellular ligand, transmembrane receptors, and a multitude of intracellular proteins, has been termed a Wnt ‘signalosome’. Wnt signalosomes have one basic purpose: the inhibition of β-catenin phosphorylation, allowing β-catenin to become resistant to proteosomal degradation. As a result, β-catenin can accumulate and spread throughout the cell. Most importantly, β-catenin binds to a number of transcription factors, and via transcriptional co-factor activity, modulates the expression of downstream genes. The best described targets of β-catenin are T-cell transcription factor/lymphoid enhancer binding factor family transcription factors, although other unrelated transcription factors can also be regulated by β-catenin. The mechanism by which β-catenin phosphorylation is repressed in signalosomes is not clear, but appears to involve two processes. Firstly, the intracellular C-termini of LRP5/6 co-receptors are able to compete with β-catenin as phosphorylation substrates for GSK3β. Secondly, signalosome formation triggers internalization of the entire complex from the plasma membrane into the endosomal system. This complex continues to signal from the cytosolic face of intracellular membranes until it is eventually sequestered into multi-vesicular bodies. It is believed that this removes the canonical Wnt signaling pool of GSK3β from the cytosol, which in turn protects newly synthesized β-catenin from proteosomal targeting (Dobrowolski and De Robertis, 2012) (Figure 1).

## The importance of Wnt signaling to higher brain function

A key role for Wnt cascades during embryogenesis is well established. It is thus unsurprising that Wnt signaling has been implicated as a master regulator of neurogenesis including adult neurogenesis (Zhang et al., 2011). Nonetheless, recent advances indicate a critical requirement for these signal transduction mechanisms in regulating the function of mature neurones. These roles of Wnt signaling range from supporting pre- and postsynaptic functioning to transcriptional regulation and have been reviewed in detail by others (Inestrosa and Arenas, 2010; Maguschak and Ressler, 2012; Wisniewska, 2013). Predictably therefore, behavioural and cognitive defects have been reported in adult mice with genetically modified Wnt signaling components (Maguschak and Ressler, 2012; Wisniewska, 2013). Importantly, Wnt signaling appears to be vital for both neuronal survival and regulation of higher brain function in adults. Therefore deregulation of these cascades is suggested to contribute to the pathogenesis of neurological and psychiatric diseases.

Wnt pathways appear to have particular importance in specific brain regions. For example, the canonical pathway has been reported to upregulate expression of the Ca^2+^ channel Cav3.1 in the thalamus, leading to enhanced T-type Ca^2+^ currents (Wisniewska et al., 2010). Roles in adult neurogenesis also suggest an acute requirement for Wnt signaling in proliferative regions such as the subventricular zone (Lie et al., 2005). However, an abundance of data suggests a critical requirement of Wnt signaling for the normal function of midbrain dopaminergic neurones. The number of genes encoding Wnt signaling proteins shown to be required for the normal development of this brain region in studies of transgenic mice models is remarkable (Table 1). This vast amount of data is reviewed comprehensively elsewhere (Castelo-Branco and Arenas, 2006; Alves dos Santos and Smidt, 2011). Importantly, evidence of perturbed Wnt signaling has also been reported in adults with diseases associated with dopaminergic neuronal dysfunction. For example, genes encoding a number of Wnt components have been linked to schizophrenia (De Ferrari and Moon, 2006; Mao et al., 2009; Wisniewska, 2013), while, as we outline below, hereditary forms of PD are also clearly connected to deregulated Wnt signaling (Berwick and Harvey, 2012b).

**Table 1:** Genes linked to both Wnt signaling and midbrain dopaminergic development

Protein	Description	Key reference
WNT1	Canonical Wnt ligand required for dopaminergic development in mouse.	Castelo-Branco et al. (2003)
WNT3A	Canonical Wnt ligand required for dopaminergic development in mouse.	Castelo-Branco et al. (2003)
WNT5A	PCP pathway ligand required for dopaminergic development in mouse.	Castelo-Branco et al. (2003)
SRFP1	Secreted Wnt modulator. Compound knockout with *SRFP2* phenocopies *WNT5A* knockout.	Kele et al. (2012)
SRFP2	Secreted Wnt modulator. Compound knockout with *SRFP1* phenocopies *WNT5A* knockout.	Kele et al. (2012)
DKK1	Secreted Wnt inhibitor. Required for midbrain differentiation.	Ribeiro et al. (2011)
FZD3	FZD receptor. Simultaneous knockout with *FZD6* causes defective midbrain morphogenesis.	Stuebner et al. (2010)
FZD6	FZD receptor. Simultaneous knockout with *FZD3* causes defective midbrain morphogenesis.	Stuebner et al. (2010)
LRP6	Wnt co-receptor. *LRP6* knockout mice display delayed dopaminergic differentiation.	Castelo-Branco et al. (2010)
Wntless	Required for Wnt ligand secretion. Knockout mice show severe mid- and hindbrain abnormalities.^a^	Carpenter et al. (2010)
β-catenin	Effector of canonical pathway. Knockout causes mid/hindbrain deformation.^a^	Brault et al. (2001)
Nurr1	β-catenin-regulated transcription factor. Required for dopaminergic development in mouse.	Zetterström et al. (1997)

^a^Knockout limited to Wnt1-expressing cells via Wnt1-CRE-driven recombination.

Thus Wnt signaling pathways appear to be important regulators of neuronal function throughout life, and deregulation is likely to play a major role in the pathogenesis of neurological diseases. Sufficient data exist to suggest a particular requirement for well-regulated Wnt signaling within the dopaminergic neurones lost in PD. As we argue below, this makes the growing evidence of a connection between Wnt signaling and familial forms of PD particularly intriguing.

## Familial PD genes and Wnt signaling

In the vast majority of cases PD is an idiopathic condition. Although a number of environmental risk factors have been identified, such as exposure to certain pesticides, the major determining factor is age. Nonetheless, our understanding of PD has changed dramatically in recent years with the discovery of gene mutations that can cause the disease. Genes identified at genetic loci previously identified in linkage studies of individuals with familial PD are often referred to as *PARK* genes. Nonetheless, additional genes and loci have been identified in studies of individual families and genome-wide association studies. Below, we summarize the evidence linking a striking number of these genes to canonical Wnt signaling (summarized in Table 2) and argue a persuasive case for a key role of deregulated Wnt cascades in the pathogenesis of PD.

**Table 2:** Genes linked to both Wnt signaling and PD

Protein	Description	Key reference
LRRK2	Product of *PARK8.* Binds various canonical Wnt proteins and modulates pathway activity.	Berwick and Harvey (2012a)
Parkin	Product of *PARK2.* Represses β-catenin.	Rawal et al. (2009)
VPS35	Product of *PARK17*. Required for Wnt secretion.	Deng et al. (2013)
Nurr1	β-catenin effector. Loss of *Nurr1* associated with PD pathology and motor dysfunction in mice.	Kitagawa et al. (2007)
GSK3β	Central canonical Wnt protein. Polymorphisms influence PD risk.	Kwok et al. (2005)
WNT3	Wnt ligand. Possible PD risk locus in Ashkenazi Jewish populations.	Liu et al. (2011)

### LRRK2

Mutations in *LRRK2* are found at the PARK8 locus, and have been identified as a cause of a familial Parkinson's. *LRRK2* encodes a member of the Roco family of proteins called leucine-rich repeat kinase 2 (LRRK2) (Paisán-Ruíz et al., 2004; Zimprich et al., 2004). Intriguingly, although beyond the scope of this review, LRRK2 has also been associated with cancer, leprosy, and Crohn's disease (Hassin-Baer et al., 2009; Van Limbergen et al., 2009; Zhang et al., 2009). LRRK2 mutations contribute to 1%–5% of PD cases worldwide, which represents the greatest contribution from any known cause, environmental or genetic (Kumari and Tan, 2009). It has been suggested that the study of LRRK2 function may be especially informative for PD, since patients with LRRK2 mutations display clinical symptoms that are indistinguishable from idiopathic PD, and post-mortem brain pathologies are also largely identical (Zimprich et al., 2004). In addition to these classical PD traits, post-mortem pathology has revealed an increased tendency of PD patients with *LRRK2* mutations to display aggregates of tau protein (Zimprich et al., 2004; Khan et al., 2005; Rajput et al., 2006; Ujiie et al., 2012). Tau pathology is usually considered a feature of Alzheimer's disease (AD) (Inestrosa et al., 2012). These observations have led to the suggestion that LRRK2 might function upstream of signaling pathways contributing to the pathogenesis of multiple forms of neurodegeneration (Taymans and Cookson, 2010).

LRRK2 is a complex 2527 amino acid protein, containing both kinase and GTPase activities as well as a number of protein–protein interaction domains. LRRK2 GTPase activity is conferred by the presence of adjacent Roc (Ras of complex proteins) and COR (C-terminal of Roc) domains, which together can be considered a single RocCOR tandem domain since Roc and COR domains are always expressed together in nature (Marín et al., 2008). Curiously, extensive research has failed to identify a reproducible kinase substrate for LRRK2, although a very wide range of interacting proteins have been found, suggesting that LRRK2 may function primarily as a scaffolding protein (Berwick and Harvey, 2011; Lewis and Manzoni, 2012). Both the kinase and GTPase activities of LRRK2 do appear important for function however. Although amino acid variations have been identified throughout the LRRK2 protein sequence, the only changes proven to segregate with PD come from mutations in exons encoding these enzymatic activities. At the level of protein function, LRRK2 has been linked to a number of cellular processes, including autophagy, vesicle trafficking, and microtubule dynamics. However, the precise roles of this protein remain unclear.

Via interactions with a number of established Wnt signaling components, LRRK2 has been strongly implicated as a key scaffold protein in the canonical pathway. Initial work using yeast two-hybrid, co-immunoprecipitation, and confocal microscopy reported a direct interaction between LRRK2 and all three mammalian DVL proteins (Sancho et al., 2009). In light of the central role of DVL proteins in all major branches of Wnt signaling, this observation could be of great importance. The interaction surfaces were mapped to the LRRK2 RocCOR tandem domain and the dishevelled–Egl10–pleckstrin domain of DVL1-3, and perhaps predictably, LRRK2 mutations within the RocCOR domain were found to modulate interaction strength (Sancho et al., 2009). Functional follow-up studies used TOPflash assays (Veeman et al., 2003) to quantify β-catenin transcriptional activity (i.e. canonical Wnt signaling). Co-transfection of LRRK2 protein with DVL1-3 increased DVL-driven canonical Wnt activity (Berwick and Harvey, 2012a). Importantly, the LRRK2 mutations R1441C, Y1699C, and G2019S, which reside within the Roc, COR, and kinase domains, respectively, were all found to weaken this effect. Furthermore, artificial mutations to the Roc and kinase domain that rendered LRRK2 GTPase- or kinase-dead were also found to be inhibitory, as was the pharmacological inhibition of LRRK2 kinase activity. Clearly, the implications of mutations throughout LRRK2 all inhibiting canonical Wnt signaling are potentially of vast significance.

The capacity for LRRK2 to enhance DVL1-driven Wnt signaling increased further by targeting LRRK2 to membranes. In agreement with this, LRRK2 was discovered to bind directly to the intracellular domain of the LRP6 membrane receptor, while treatment of HEK293 cells with recombinant Wnt3a increased the amount of endogenous LRRK2 protein present in membrane fractions. These observations are consistent with the Wnt signalosome hypothesis and suggest that LRRK2 affects canonical pathway activity at cellular membranes (Berwick and Harvey, 2012a). Similar to the interaction with DVL proteins, the binding of LRRK2 to LRP6 was found to require the LRRK2 RocCOR tandem domain (Berwick and Harvey, 2012a). Moreover, quantitative yeast two-hybrid assays revealed the strength of the LRRK2–LRP6 interaction to be weakened by the R1441C, R1441G, and Y1699C LRRK2 mutations. Taken together, these data suggest a model where in response to Wnt ligand, LRRK2 is recruited to cellular membranes where, via interaction with DVL proteins and LRP6, this protein plays an important role in signalosome formation.

Surprisingly, siRNA-mediated knockdown of LRRK2 was also found to enhance canonical Wnt signaling (Berwick and Harvey, 2012a). In agreement with this, murine embryonic fibroblasts derived from *LRRK2* knockout mice also display enhanced canonical Wnt activity compared with wild-type controls (unpublished data). Importantly, the effect of knocking down LRRK2 expression was observed under Wnt3a- and DVL1-stimulated conditions but also basally, suggesting an inhibitory role for LRRK2 in the β-catenin destruction complex. Consistent with this idea, co-immunoprecipitation of endogenous LRRK2 from mouse brain revealed LRRK2 to exist in complex with multiple components of the β-catenin destruction complex, including Axin1, GSK3β, and β-catenin (Berwick and Harvey, 2012a). It should be noted that an interaction between LRRK2 and GSK3β has been reported previously in *Drosophila* (Lin et al., 2010). Intriguingly, interaction strength was reported to be enhanced by the G2019S *LRRK2* mutation (Lin et al., 2010). Thus, not only does LRRK2 interact with numerous components of the canonical pathway, but the strength of interaction with at least three of these is affected by PD-causing *LRRK2* mutations.

Taken together, these data suggest a central role for LRRK2 in canonical Wnt signaling. Under basal conditions, LRRK2 forms part of the cytosolic β-catenin destruction complex. Loss of LRRK2 compromises this role, leading to disruption of the complex and pathway activation. Following stimulation with Wnt ligand, LRRK2 is recruited to membranes. Here, via interaction with DVL proteins, the β-catenin destruction complex and LRP6, LRRK2 assists in the formation of Wnt signalosomes, enhancing the activity of the canonical Wnt pathway. Familial *LRRK2* mutations decrease the ability of LRRK2 to enhance Wnt signaling activity.

### Parkin

The E3 ubiquitin ligase Parkin is encoded by the *PARK2* gene. In contrast to *LRRK2*, *PARK2* mutations represent a relatively rare cause of PD, are inherited in an autosomal recessive manner, and are considered loss-of-function mutations (Gasser, 2009). Patients display an atypical form of PD, with early onset of motor symptoms and a brain pathology that is largely restricted to loss of dopaminergic neurones (Gasser, 2009). PD caused by mutations in *PARK2* has been suggested to have much in common with other early onset autosomal recessive forms of parkinsonism caused by mutations in *PINK1*/*PARK6* or *DJ-1*/*PARK7*. Perhaps unsurprisingly, these autosomal recessive PD genes appear to function in a common process, namely the regulation of mitophagy (autophagy of mitochondria), and also interact genetically in animal models (Dodson and Guo, 2007). However, the control of mitophagy is only one aspect of Parkin function, as this protein contributes to other cellular processes that when deregulated might contribute to neurodegeneration.

Interestingly, Parkin has been reported to induce β-catenin ubiquitination and degradation, thereby repressing canonical Wnt activity (Rawal et al., 2009). Concordantly, Parkin dysfunction triggers an accumulation of β-catenin leading to up-regulation of canonical Wnt signaling. Rawal et al. (2009) showed that β-catenin protein levels, but not mRNA levels, were increased significantly in the ventral midbrain of Parkin knockout mice compared with wild-type controls, but intriguingly, not in other brain regions. Since Wnt ligands are generally considered neuroprotective, increased canonical Wnt signaling might seem a counter-intuitive pathomechanism for PD. However, elevated Wnt signaling leading to attempted cell-cycle re-entry in postmitotic neurones is detrimental and has been reported in both AD brains and AD mouse models (Currais et al., 2009). Consistent with this, Parkin knockout mice were found to display increased levels of cyclin E (Rawal et al., 2009). As such, one physiological role of Parkin function might be to protect dopaminergic neurones from excessive canonical Wnt signaling, particularly in brain regions implicated in the pathogenesis of PD (Rawal et al., 2009). These observations support the idea that Wnt signaling needs to be regulated within well-defined boundaries, and also add weight to the notion that the canonical Wnt pathway is particularly important in midbrain dopaminergic neurones.

### VPS35

Vacuolar sorting protein 35 homologue (VPS35) is the product of the *VPS35*/*PARK17* gene, and was identified as a cause of autosomal dominant PD in 2011 (Vilariño-Güell et al., 2011; Zimprich et al., 2011). The overall contribution of VPS35 mutations to PD is thought to be low (Deng et al., 2013). However, several lines of evidence suggest that the study of VPS35 protein function will be particularly informative. Firstly, similar to LRRK2, patients with mutations in *VPS35* display symptoms that are very similar to idiopathic PD, including late age of onset. Indeed, *VPS35* mutations are reckoned to be the second major genetic cause of late onset PD after LRRK2 (Deng et al., 2013). Secondly, VPS35 has been linked previously to AD. For example, the levels of VPS35 protein are decreased in post-mortem brains of AD patients (Small et al., 2005), while mice lacking VPS35 show behavioural and pathological features consistent with this disorder (Wen et al., 2011). In this regard, VPS35 is also similar to LRRK2, since ‘tau pathology’, usually a post-mortem hallmark of AD, has been described in patients (Zimprich et al., 2004; Khan et al., 2005; Rajput et al., 2006; Ujiie et al., 2012) and animal models harbouring *LRRK2* mutations (Li et al., 2009; Lin et al., 2010; Melrose et al., 2010). Thus it is interesting to speculate that LRRK2 and VPS35 might function in a common pathway that is required for neuronal survival across the brain, but is especially vital in brain regions damaged in idiopathic PD.

VPS35 is well described as a key component of the retromer complex, important for the sorting and retrograde transport of membrane-localized proteins from endosomes to the *trans*-Golgi network (Seaman, 2012). Intriguingly, VPS35 has been tied to Wnt signaling in a number of studies. Initial work published in 2006 described a requirement of the retromer complex for Wnt signaling during embryogenic patterning in the model organism *Caenorhabditis elegans* (Coudreuse et al., 2006; Prasad and Clark, 2006). Providing a mechanistic interpretation for these observations, a number of groups subsequently reported that loss of VPS35 function prevents the endosome-to-Golgi recycling of Wntless, a protein essential for secretion of Wnt ligands (Belenkaya et al., 2008; Franch-Marro et al., 2008; Port et al., 2008; Kim et al., 2009). This interaction between VPS35 and Wntless is particularly fascinating in the context of PD, as mice lacking Wntless display profound developmental abnormalities of the midbrain (Carpenter et al., 2010). The only established PD-causing mutation in the *VPS35* gene encodes a D620N amino acid substitution (Deng et al., 2013). We are unaware of any studies investigating the effect of D620N on Wnt signaling. Nonetheless, we speculate that this mutation might impair VPS35 function causing a gradual accumulation of Wntless within the endosomal pathway subsequently leading to decreased Wnt secretion and loss of Wnt signaling activity in neighbouring neurones. If this hypothesis is correct, both mutations in *LRRK2* and *VPS35* would contribute to the pathogenesis of PD by decreasing Wnt signaling activity.

### Nurr1

The transcription factor nuclear receptor-related protein 1 (Nurr1), encoded by *NR4A2*, is a well-established key regulator of dopaminergic neuronal development during embryogenesis, and important for the expression of genes regulating dopaminergic neurotransmission (Arenas, 2005; Jankovic et al., 2005). Even though sequence variations within *NR4A2* are not considered a widespread genetic cause of PD (Nichols et al., 2004; Tan et al., 2004), one mutation leading to decreased Nurr1 expression has been identified in a PD patient (Sleiman et al., 2009), and decreased Nurr1 expression has also been reported in midbrain dopaminergic neurones from patients with idiopathic PD (Jankovic et al., 2005). In addition, Nurr1 knockout in midbrain dopaminergic neurones of adult mice results in neuronal degeneration and impaired motor function (Kadkhodaei et al., 2009). Nurr1 has also been reported to regulate the expression of α-synuclein, the major component of Lewy bodies, and the identified *NR4A2* mutation increases the expression of α-synuclein (Yang and Latchman, 2008). Therefore, impairment of Nurr1 activity is likely to impact upon PD progression partially via upregulation of α-synuclein expression. Importantly, Nurr1 has been reported to interact with β-catenin on promoters of Wnt and Nurr1 responsive target genes (Kitagawa et al., 2007). In particular, β-catenin and Nurr1 were shown to transactivate the KCNIP4 promoter (Kitagawa et al., 2007). The possibility that Nurr1 and Wnt signaling pathways are able co-activate genes important for neurodegeneration is a tantalizing prospect.

### GSK3β

GSK3β is well established as a critical component of the canonical Wnt signaling pathway (Maiese et al., 2008; Freese et al., 2010; Hur and Zhou, 2010; Taelman et al., 2010; Kim et al., 2011). The gene encoding this protein (*GSK3B*) has been implicated in PD risk in two genetic studies (Kwok et al., 2005; Kalinderi et al., 2011), although a third found no association (Wider et al., 2011). Therefore, GSK3β may only contribute to PD risk in certain populations. GSK3 was first identified as a kinase phosphorylating glycogen synthase (Woodgett and Cohen, 1984). The two isoforms, GSK3α and GSK3β, are ubiquitously expressed in mammalian cells and are considered constitutive kinases, i.e. they are inhibited by activation of upstream signaling pathways, but are active under basal conditions (Hur and Zhou, 2010; Kaidanovich-Beilin and Woodgett, 2011; Kim et al., 2011). As might be expected, GSK3α and GSK3β display some redundancy of function. However, isoform-specific functions are probable, and thus the ratio and absolute expression levels of these proteins vary between cell types. Indeed, a brain-specific GSK3β splice-form has been described, which is believed to play a specific role in neuronal differentiation (Mukai et al., 2002; Wood-Kaczmar et al., 2009; Castaño et al., 2010).

GSK3β can be modulated by a surprisingly large number of upstream signaling pathways. In addition to the canonical Wnt cascade, pathways affecting GSK3β activity include phosphatidylinositol 3′ kinase, notch and hippo (Kaidanovich-Beilin and Woodgett, 2011). The requirement of one protein to function in so many pathways, while still retaining signal specificity, has led to the suggestion that GSK3β almost certainly exists in distinct sub-cellular pools. A number of neuronal GSK3β substrates have been reported, including proteins implicated in synaptic plasticity, such as dynamin-1 and gephyrin (Clayton et al., 2010; Tyagarajan et al., 2011), and transcription factors, for example cAMP response element binding protein (Grimes and Jope, 2001). However, it is perhaps most interesting to note the number of microtubule associated proteins (MAPs) that have been implicated as GSK3β substrates. These include tau, collapsing response mediator protein 2, and MAP1B (Mandelkow et al., 1992; Cole et al., 2004; Trivedi et al., 2005). Thus, GSK3β appears to be a master regulator of microtubule dynamics in neurones. Since GSK3β is a LRRK2 interacting protein (Lin et al., 2010; Berwick and Harvey, 2012a) and LRRK2 was found in association with microtubules and affects tau phosphorylation (Zimprich et al., 2004; Khan et al., 2005; Biskup et al., 2006; Gloeckner et al., 2006; Rajput et al., 2006; Gandhi et al., 2008; Gillardon 2009a, b; Sancho et al., 2009; Dzamko et al., 2010; Lin et al., 2010; Kawakami et al., 2012; Kett et al., 2012; Sheng et al., 2012; Ujiie et al., 2012), LRRK2 and GSK3β might form a Wnt ligand-regulated complex governing microtubule dynamics in neurones. *MAPT*, the gene encoding tau, has also been implicated as a major genetic determinant of PD (Golub et al., 2009; González-Pérez et al., 2009; Satake et al., 2009; Simón-Sánchez et al., 2009). Thus, these connections allow for the possibility that mutations in *LRRK2*, *GSK3B*, or *MAPT* all result in neurodegeneration via affecting microtubule function.

### WNT3

The *WNT3* gene encodes a conserved canonical Wnt ligand and is located on chromosome 17 at 17q21, within a 1.8 Mb region of linkage disequilibrium, centred on the *MAPT* gene (Caffrey and Wade-Martins, 2007; Kalinderi et al., 2009; Liu et al., 2011). In most human populations this genomic region forms a so-called *MAPT* H1 haplotype, but among Caucasian populations an alternative *MAPT* H2 haplotype can be found (Caffrey and Wade-Martins, 2007; Kalinderi et al., 2009). There is little recombination within this region due to the presence of a 900 kb inversion within the centre of the *MAPT* H2 haplotype (Hardy et al., 2005). The *MAPT* H1 haplotype has been repeatedly found to confer a greater risk of PD in genome-wide association studies (Golub et al., 2009; González-Pérez et al., 2009; Satake et al., 2009; Simón-Sánchez et al., 2009). It has been suggested that variations within the *MAPT* gene itself confer some or most of the *MAPT* H1 risk. However, since the genes within the disequilibrium region all co-segregate with *MAPT*, they are all potential risk factors. Intriguingly, a detailed study of the *MAPT* H1 region in Ashkenazi Jews found linkage with multiple regions within the region, including linkage to *WNT3* (Liu et al., 2011). This research is at early stages, and we note that no linkage to *WNT3* could be found in Russian PD patients (Filatova et al., 2011). Nonetheless, the possibility that altered function of a Wnt ligand might affect PD risk is an exciting possibility, and has clear implications for potential treatments.

## Additional connections between canonical Wnt signaling and neurodegeneration

The presented genetic links between PD and Wnt signaling make a powerful case for a role of canonical Wnt pathway deregulation in the molecular aetiology of the disease. In addition, Wnt signaling is deregulated in numerous animal models of toxin-induced PD (L'Episcopo et al., 2011b; Dun et al., 2012; Gollamudi et al., 2012), and in brains of PD patients (Cantuti-Castelvetri et al., 2007). These observations speak for a potential causative role and/or role in disease progression for perturbed Wnt signaling. For example, Wnt signaling has been shown to be central to brain inflammation and adult neurogenesis (Lie et al., 2005; Berwick and Harvey, 2013; Marchetti and Pluchino, 2013). In rodent models, reactive astrocytes and microglia have been reported to protect dopaminergic neurones by activating canonical Wnt signaling, and also by promoting neurogenesis of progenitor cells originating from the subventricular zone, via a mechanism partially based on the interaction between inflammation and canonical Wnt signaling (L'Episcopo et al., 2011a, b, 2012; Marchetti and Pluchino, 2013).

Interestingly, deregulated Wnt signaling pathways are also suggested pathomechanisms for a number of additional neurological conditions, including AD, autism, and schizophrenia (see Table 3) (De Ferrari and Moon, 2006; Inestrosa and Toledo, 2008; Berwick and Harvey, 2012b; Kalkman, 2012). It is worth observing that lithium, a pharmacological treatment for psychotic disorders, is a well-established GSK3β inhibitor, i.e. this treatment might involve canonical Wnt activation (Wisniewska, 2013). The evidence linking AD to Wnt signaling defects has resulted in a unifying hypothesis for AD aetiology centred around deregulated Wnt cascades (Inestrosa and Arenas, 2010). Importantly, post-mortem brains from AD patients exhibit increased levels of GSK-3β, as well as greater β-catenin phosphorylation, indicative of decreased canonical Wnt activity (Caricasole et al., 2004; Inestrosa and Arenas, 2010). Suggesting a plausible explanation for this, Dickkopf-1 (Dkk1), a secreted protein that inhibits the canonical pathway by binding to LRP6, was also found to be elevated in AD brains (Caricasole et al., 2004; Inestrosa and Arenas, 2010). Supporting this idea, Dkk1-neutralizing antibodies protect *ex vivo* mouse brain slices from synaptic loss caused by Aβ oligomers (Purro et al., 2012). Taken together, these data suggest a model where Dkk1 inhibits Wnt signaling at the cell membrane, leading to enhanced cellular GSK-3β activity and increased repression of β-catenin. Since GSK-3β is considered the major kinase phosphorylating tau *in vivo*, this model also explains one of the major pathological hallmarks of AD: hyperphosphorylated tau. Further strengthening this hypothesis, *GSK3B* variants have been suggested as a genetic risk factor for AD (Kwok et al., 2008). The connections between LRRK2, GSK3β, and tau in PD are intriguing but clearly provide a valid pathomechanism underlying AD. Furthermore, an LRP6 variant conferring reduced Wnt signaling is associated with AD in carriers of the ApoE4 risk variant in genome-wide association studies (De Ferrari et al., 2007). Interestingly, LRP6 is not just a direct interactor of LRRK2 (Berwick and Harvey, 2012a); VPS35 and ATP13A2 (the product of the *PARK9* gene) have also been reported to bind the LRP6 intracellular domain (George et al., 2007; Usenovic et al., 2012). These observations require follow-up studies, but if correct, mean that a single canonical Wnt protein, itself a risk factor for neurodegeneration, physically interacts with the products of no less than three *PARK* genes.

**Table 3:** Genes linked to Wnt signaling and other neurological diseases

Protein	Description	Key reference
WNT2	Wnt ligand. Linked to autism.	Marui et al. (2010)
FZD3	FZD receptor. Linked to schizophrenia in Asian populations.	Katsu et al. (2003)
FZD9	FZD receptor. Linked to autism.	Sanders et al. (2011)
LRP6	Wnt co-receptor. Modulates risk conferred by *APOE* isoforms in AD.	De Ferrari et al. (2007)
DISC1	Schizophrenia risk gene. *DISC1* activates canonical Wnt signaling via inhibition of GSK3β.	Mao et al. (2009)
GSK3β (I)	Central canonical Wnt component. Linked to genetic risk of AD.	Kwok et al. (2008)
GSK3β (II)	Central canonical Wnt component. Implicated in bipolar disorder as a target of lithium.	Wisniewska (2013)

## Future research

It is clear that Wnt signaling pathways are of fundamental importance to the function of adult neurones. This requirement appears particularly acute for the dopaminergic neurones of the ventral midbrain, and changes in established Wnt components appear to influence PD risk. *Vice versa*, compelling genetic and functional data indicate a number of *PARK* genes as important modulators of the canonical Wnt pathway. In addition, Wnt signaling is undoubtedly central to processes that are critical for PD progression, such as neuroinflammation, adult neurogenesis, microtubule stability, axonal function, and membrane trafficking (Inestrosa and Arenas, 2010; Berwick and Harvey, 2011; Marchetti and Pluchino, 2013). Thus, we contend that the evidence supporting a key role for perturbed canonical Wnt signaling in the aetiology of PD is both plausible and persuasive.

Despite this, a great deal of research remains to be carried out. Most notably, there is no direct evidence that loss of canonical Wnt signaling in the adult brain is sufficient to elicit PD. Nonetheless, continuing genome-wide association studies may provide additional links to Wnt signaling. Suggested associations require further investigation. In particular, linkage of PD to the *WNT3* locus needs investigation in different populations. By contrast, *GSK3B* variants do appear to modulate PD risk in the context of additional genetic cues, but probably not in all populations. Importantly, the effects of this genetic association need detailed investigation in all signaling pathways involving GSK3β.

Further examinations of post-mortem PD brains might be useful, and have already garnered evidence of altered canonical Wnt signaling (Cantuti-Castelvetri et al., 2007). However, patient samples invariably come from late-stage PD, where evidence of initial aetiology will largely be masked by the more profound changes caused by cell death. Instead, we anticipate more conclusive data from animal models of early/preclinical stages of PD. Genes encoding canonical Wnt signaling proteins are required for the development of midbrain dopaminergic neurones (Table 1), but the functions of these genes in neurodegeneration are less clear. Therefore, the use of inducible conditional knockout/knock-in rodent models, or viral gene transduction to modify protein expression in adult rodents, might be more informative (Wilkinson et al., 2011). These animals could be tested for motor, cognitive, behavioural, and electrophysiological defects, and tissue investigated for neurodegenerative changes. Here, the investigator would encounter the difficulties of recapitulating PD in an organism that rarely lives beyond 2 years of age, and displays little evidence of neurodegeneration under ordinary conditions. Nonetheless, we are confident that with the right experimental design this approach can be fruitful. For example, conditional knockout mice could be treated with low doses of toxins (i.e. 6-hydroxydopamine or 1-methyl-4-phenyl-1,2,3,6-tetrahydropyridine) to provide an additional ‘environmental’ stress triggering a more profound phenotype.

As a final observation, we should highlight the potential implications of a causal role for altered canonical Wnt signaling in PD aetiology for the treatment of this condition. In particular, the development of targeted methods for regulating Wnt signaling might be considered as a strategy for new therapies in the future (Inestrosa and Arenas, 2010). Even if such treatments fail to cure the disease, one might at least expect enhanced Wnt signaling to support adult neurogenesis and normal synaptic function. In addition, modulation of inflammatory responses by Wnt signaling would also be predicted to improve symptoms. It is also worth noting that one therapeutic strategy currently under development—implantation of dopaminergic neuronal precursors derived from human stem cells—is crucially dependent on Wnt ligands for programming and specification (Kriks et al., 2011).

In conclusion, evidence for a role of deregulated canonical Wnt signaling as an important cause of familial and idiopathic PD is accumulating. Nonetheless, this hypothesis needs further investigation in adult animal models. We stress that targeting Wnt cascades at different points in the signaling cascade represents a promising therapeutic approach for modulating PD progression. It is therefore crucial that we acquire more knowledge of the specific pathways involved in the pathogenesis of the disease. Key remaining questions include: What is the exact role of *PARK* genes? And, which Wnt receptors might be promising therapeutic targets for small molecules?

## Funding

We are grateful for the support of our work on PD and Wnt signaling by the Wellcome Trust (WT088145MA, WT095010MA) and the Michael J Fox Foundation.

**Conflict of interest:** none declared.
